# Impact of HER2-Low Expression on Clinical Outcomes in Metastatic Breast Cancer Treated with CDK4/6 Inhibitors

**DOI:** 10.3390/jcm15051898

**Published:** 2026-03-02

**Authors:** Şahin Bedir, Tanju Kapagan, Burçin Çakan Demirel, Merve Tokocin, Çiğdem Yıldırım, Semra Taş, Yakup Bozkaya, Abdilkerim Oyman, Nilufer Bulut, Gökmen Umut Erdem

**Affiliations:** 1 Department of Medical Oncology, Faculty of Medicine, Istinye University, Gaziosmanpaşa Hospital, Istanbul 34250, Turkey; dr_yakupbozkaya@hotmail.com (Y.B.); droyman84@gmail.com (A.O.); 2 Department of Medical Oncology, University of Health Sciences, Basaksehir Cam and Sakura City Hospital, Istanbul 34480, Turkey; tanjukapagan2016@gmail.com (T.K.); ferlut@gmail.com (N.B.); gokmenumut@hotmail.com (G.U.E.); 3 Department of Medical Oncology, Istanbul Medipol University, Medipol Mega University Hospital, Istanbul 34214, Turkey; burcin.cakandemirel@gmail.com; 4 Department of General Surgery, University of Health Sciences, Bagcilar Training and Research Hospital, Istanbul 34100, Turkey; mervetokocin@gmail.com; 5 Department of Medical Oncology, University of Health Sciences, Samatya Training and Research Hospital, Istanbul 34098, Turkey; drcigdemyil@hotmail.com; 6 Department of Medical Oncology, Faculty of Medicine, Pamukkale University Hospital, Denizli 20360, Turkey; semratasdr@gmail.com

**Keywords:** breast cancer, CDK 4/6 inhibitors, endocrine therapy, HER2-low, HER2-zero

## Abstract

**Background:** The prognostic significance of low-level human epidermal growth factor receptor 2 (HER2) expression in hormone receptor-positive/HER2-negative (HR+/HER2−) metastatic breast cancer remains unclear, particularly in patients treated with cyclin-dependent kinase 4/6 inhibitors (CDK4/6i). This study aimed to evaluate the impact of HER2-low status on treatment response and survival outcomes in this setting. **Methods:** This multicenter retrospective cohort study included patients with HR+/HER2− metastatic breast cancer who received first-line endocrine therapy combined with palbociclib or ribociclib between January 2018 and May 2025. HER2-low tumors were defined as immunohistochemistry (IHC) 1+ or 2+ with negative in situ hybridization, while HER2-zero tumors were classified as IHC 0. Treatment response, progression-free survival (PFS), and overall survival (OS) were compared between groups using Kaplan–Meier analysis and Cox regression models. **Results:** A total of 309 patients were analyzed, including 122 (39.5%) with HER2-low disease and 187 (60.5%) with HER2-zero disease. Baseline clinicopathological characteristics were well balanced between groups. The overall response rate was 75.4% in the HER2-low group and 72.7% in the HER2-zero group (*p* > 0.05). Median PFS was 23.9 months for HER2-low patients and 25.2 months for HER2-zero patients (log-rank *p* = 0.785). Median OS was 49.5 and 53.1 months, respectively, with no statistically significant difference (log-rank *p* = 0.649). HER2 status was not an independent predictor of PFS or OS in multivariable analyses. **Conclusions:** In patients with HR+/HER2− metastatic breast cancer treated with first-line endocrine therapy plus CDK4/6 inhibitors, HER2-low expression was not associated with differences in treatment response or survival outcomes. These findings suggest that HER2-low status does not have prognostic or predictive relevance in this endocrine-sensitive population.

## 1. Introduction

Breast cancer (BC) remains the most frequently diagnosed malignancy among women worldwide and represents a major contributor to cancer-related morbidity and mortality [1]. From a molecular standpoint, breast cancer is categorized into four distinct subtypes based on the expression of the human epidermal growth factor receptor 2 (HER2) and hormone receptors (HRs). This molecular classification plays a crucial role not only in predicting clinical outcomes but also in guiding the selection of targeted therapeutic approaches [2].

The HR+/HER2− subtype represents the most prevalent molecular category of breast cancer, accounting for approximately 65–75% of invasive cases [3]. Among these patients, an estimated 5–10% present with de novo metastatic disease at diagnosis. Additionally, it has been reported that about 20–30% of individuals initially diagnosed at an early stage eventually develop distant metastases during follow-up [4].

In patients with hormone receptor–positive/human epidermal growth factor receptor 2–negative metastatic breast cancer (MBC), the combination of endocrine therapy (ET) with cyclin-dependent kinase 4/6 inhibitors (CDK4/6i) is currently regarded as the standard first-line therapeutic approach [5]. The introduction of CDK4/6 inhibitors into clinical practice has markedly improved survival outcomes in this population, leading to a significant shift in the treatment landscape. Nevertheless, the emergence of resistance to these agents and the ongoing need to identify reliable biomarkers capable of predicting treatment response remain major clinical challenges [6].

According to the 2018 guidelines of the American Society of Clinical Oncology (ASCO) and the College of American Pathologists (CAP), approximately 80% of breast tumors without HER2 protein overexpression are classified as HER2-negative (HER2−). Among these cases, nearly 45–55% exhibit a HER2 immunohistochemistry (IHC) score of 1+ or 2+ with a negative in situ hybridization (ISH) result, a subgroup referred to as HER2-low breast cancer [7,8].

Interest in the HER2-low subgroup has grown substantially, particularly following the groundbreaking findings of the DESTINY-Breast04 trial evaluating trastuzumab deruxtecan, an antibody–drug conjugate (ADC) [9]. Bidirectional crosstalk between HER2 and hormone receptor (HR) signaling pathways plays a critical role in the development of endocrine resistance [10]. Consequently, understanding how varying levels of HER2 expression influence clinical response and survival outcomes—especially in HR+/HER2− metastatic breast cancer patients treated with CDK4/6 inhibitors in combination with endocrine therapy—has become an important area of investigation. This study was designed to investigate the potential impact of HER2-low expression on endocrine therapy effectiveness and clinical outcomes in patients with HR+/HER2− metastatic breast cancer receiving treatment with CDK4/6 inhibitors.

## 2. Materials and Methods

### 2.1. Study Design and Patient Selection

This retrospective cohort study analyzed data from patients diagnosed with hormone receptor-positive/HER2-negative metastatic breast cancer who received a combination of CDK4/6 inhibitors (ribociclib (Novartis Pharmaceuticals, Basel, Switzerland) or palbociclib (Pfizer Inc., New York, NY, USA)) and endocrine therapy between January 2018 and May 2025. The study was conducted across three medical oncology centers.

### 2.2. Biomarker Assessment (ER, PR, and HER2)

Estrogen receptor (ER), progesterone receptor (PR), and Ki-67 were evaluated using immunohistochemistry (IHC), while HER2 status was assessed on primary tumor specimens by IHC and/or in situ hybridization (ISH). Tumors were considered hormone receptor-positive when estrogen receptor (ER) expression was ≥10% by immunohistochemistry; progesterone receptor (PR) expression was recorded descriptively [11]. HER2 immunohistochemistry was performed using validated staining platforms and antibodies at each participating center in accordance with the 2018 ASCO/CAP guidelines [12]. HER2 scoring was conducted by experienced breast pathologists, and centralized pathology re-review was not performed due to the retrospective multicenter design. HER2-low tumors were defined as those with an IHC score of 1+ or 2+ accompanied by a negative ISH result. Tumors with an IHC score of 0 were classified as HER2-zero (HER2-0).

### 2.3. Inclusion and Exclusion Criteria

**Inclusion Criteria:**

1. Histopathologically confirmed hormone receptor-positive (ER ≥10%) and HER2-negative (IHC 0, 1+, or 2+/ISH−) metastatic breast cancer.
2. Receipt of combination therapy with a CDK4/6 inhibitor (palbociclib or ribociclib) and endocrine therapy.
3. Presence of either de novo metastatic disease or secondary (acquired) endocrine resistance.
4. Availability of consistent clinical and radiological follow-up data throughout treatment.
5. A minimum follow-up duration of at least 6 months from the initiation of therapy.

**Exclusion criteria:**

1. Patients with HER2 amplification or overexpression (IHC 3+ or ISH positive).
2. Individuals diagnosed with triple-negative breast cancer.
3. Patients who previously received chemotherapy in the metastatic setting.
4. Evidence of primary endocrine resistance.
5. Cases with incomplete or insufficient pathology or follow-up data.

### 2.4. Data Collection

Demographic information (age, menopausal status), tumor characteristics (histological type, grade, HR and HER2 status), features of metastatic disease (sites of metastases), administered treatment regimens (type of CDK4/6 inhibitor, concurrent endocrine agent), as well as treatment response and survival data were retrospectively collected from patient records and electronic medical record systems.

### 2.5. Follow-Up and Survival Analysis

Patients were monitored at regular 3-month intervals following the initiation of CDK4/6 inhibitor therapy. Median follow-up time was estimated using the reverse Kaplan–Meier method. Progression and survival data were retrospectively obtained from patient records and hospital databases. Progression-free survival (PFS) was defined as the time from the start of CDK4/6 treatment to either disease progression or death. Overall survival (OS) was defined as the interval from the initiation of therapy to death or the last follow-up.

### 2.6. Statistical Analysis

All analyses were performed using SPSS version 26 (IBM Corp., Armonk, NY, USA). Continuous variables were compared using either parametric (Student’s *t*-test) or non-parametric (Mann–Whitney U) methods, following assessment of normality with the Kolmogorov–Smirnov test. Survival analyses were conducted using the Kaplan–Meier method and compared with the log-rank test. Multivariate survival analyses were performed using the Cox regression model. A *p*-value of <0.05 was considered statistically significant.

### 2.7. Ethical Approval

The study was approved by the Yeni Yüzyıl University Clinical Research Ethics Committee (Decision No: 2025/11-1685, Date: 6 November 2025) and was conducted in accordance with the principles of the Declaration of Helsinki. Patient identities were kept confidential, and only anonymized data were used [13].

## 3. Results

### 3.1. Patient Characteristics

Between January 2018 and May 2025, a total of 309 patients meeting the inclusion criteria were analyzed. Of these, 122 patients (39.5%) were classified as HER2-low (IHC 1+ or IHC 2+/ISH−) and 187 patients (60.5%) as HER2-zero (IHC 0). The median age of the entire cohort was 53 years (range, 26–90), and all patients were female. Among the patients, 217 (70.2%) were postmenopausal, and 225 (72.8%) had invasive ductal carcinoma. No significant differences were observed between the HER2-low and HER2-0 groups in terms of age, menopausal status, or tumor histology (*p* > 0.05).

Regarding hormone receptor profiles, 284 patients (92%) exhibited ER expression >50%, 233 (75.4%) had PR expression ≥ 20%, and 219 (70.9%) had Ki-67 levels ≥20%. There were no significant differences between the HER2-low and HER2-0 groups for ER expression, PR expression, or Ki-67 levels (*p* > 0.05). Additionally, de novo metastasis was present in 188 patients (60.8%), and visceral metastasis was detected in 113 patients (36.5%). No significant differences were observed between the HER2-low and HER2-0 groups regarding the rates of de novo or visceral metastases (*p* > 0.05). Key patient characteristics are summarized in Table 1.

### 3.2. Treatment Characteristics and Response Rates

A total of 214 patients (69.3%) received ribociclib-based therapy, while 95 patients (30.7%) were treated with palbociclib-based regimens. Aromatase inhibitors were administered as the accompanying endocrine therapy, and 92 patients (29.8%) also received GnRHa. The distribution of treatment did not differ significantly between the HER2-low and HER2-0 groups (*p* > 0.05).

Among the 309 patients evaluable for treatment response, the overall response rate (ORR) was 75.4% in the HER2-low group and 72.7% in the HER2-0 group, with no statistically significant difference between the two groups (*p* > 0.05). The disease control rate (DCR) was 91% in the HER2-low group and 88.8% in the HER2-0 group, also showing no significant difference (*p* > 0.05). Treatment response rates are summarized in Table 2.

### 3.3. Progression-Free Survival (PFS)

**Figure 1:**
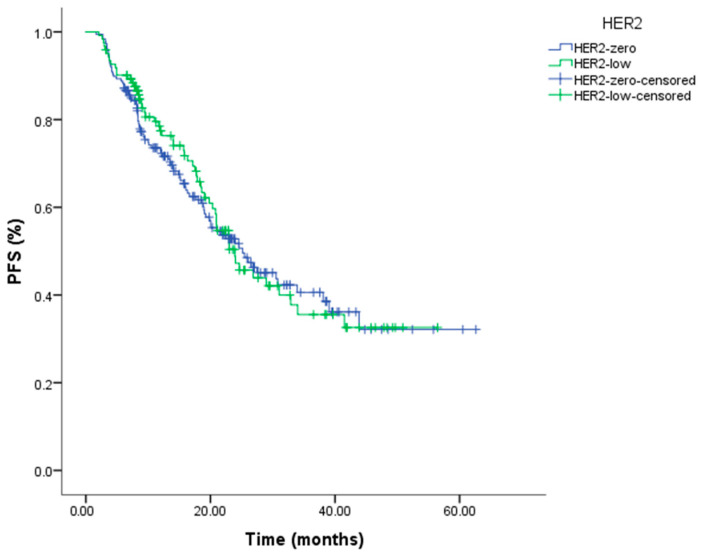
Kaplan–Meier analysis of progression-free survival according to HER2 status in HR+/HER2− metastatic breast cancer patients treated with CDK4/6 inhibitors (log-rank *p* = 0.785).

The median follow-up duration for the cohort was 22.5 months (range: 6.05–70.21). During this period, a total of 144 patients (46.6%) experienced disease progression. The median PFS for the entire cohort was 24.01 months (95% CI: 19.98–28.05). In the HER2-low group, the median PFS was 23.91 months (95% CI: 18.71–29.12), whereas it was 25.16 months (95% CI: 17.64–32.68) in the HER2-0 group (Table 3). No statistically significant difference in PFS was observed between HER2-low and HER2-0 patients (log-rank *p* = 0.785) (Figure 1).

In univariate Cox regression analysis for PFS, recurrent metastatic disease, progesterone receptor (PR) expression < 20%, Ki-67 ≥ 20%, and the presence of visceral metastases were significantly associated with shorter PFS (*p* < 0.05), whereas HER2-low expression was not significantly associated with PFS.

In multivariate Cox regression analysis, PR expression < 20%, Ki-67 ≥ 20%, and the presence of visceral metastases remained independently associated with shorter PFS (*p* < 0.05) (Table 4).

### 3.4. Overall Survival (OS)

**Figure 2:**
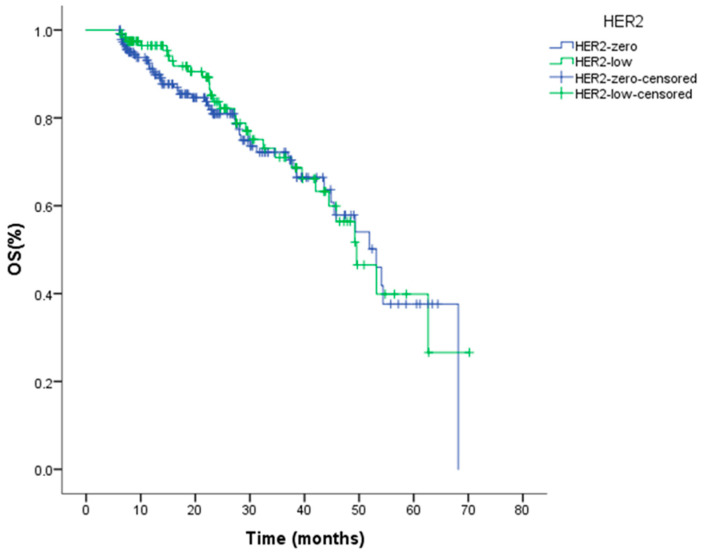
Kaplan–Meier analysis of overall survival according to HER2 status in HR+/HER2− metastatic breast cancer patients treated with CDK4/6 inhibitors (log-rank *p* = 0.649).

During the follow-up period, a total of 77 patients (24.91%) died. The median OS for the entire cohort was 51.91 months (95% CI: 45.92–57.89). Median OS was 49.54 months (95% CI: 41.82–57.26) in the HER2-low group and 53.12 months (95% CI: 44.69–61.55) in the HER2-0 group (Table 3). No significant difference in OS was observed between the two groups (log-rank *p* = 0.649) (Figure 2). Given the relatively limited number of OS events and the median follow-up duration, these OS estimates should be interpreted with caution. Nevertheless, OS findings were directionally consistent with the PFS results.

In univariate Cox regression analysis for OS, the presence of visceral metastases was significantly associated with shorter OS (*p* < 0.05), whereas HER2-low expression was not significantly associated with OS.

In multivariate Cox regression analysis, the presence of visceral metastases remained independently associated with shorter OS (*p* < 0.05) (Table 5).

### 3.5. Subgroup Analysis

In an exploratory sensitivity analysis, patients with HER2-low disease were further stratified into IHC 1+ and IHC 2+/ISH− subgroups. No statistically significant differences were observed between these subgroups in terms of ORR, PFS, or OS (*p* > 0.05).

## 4. Discussion

This study was designed to evaluate the potential impact of varying immunohistochemical HER2 expression levels on clinical outcomes in patients with HR-positive/HER2-negative metastatic breast cancer treated with first-line CDK4/6 inhibitors. In the context of targeted anti-HER2 therapies for metastatic breast cancer, the DESTINY-Breast04 and DESTINY-Breast06 trials demonstrated that trastuzumab deruxtecan (T-DXd) provides significant clinical benefit in patients with low HER2 expression, increasing the clinical relevance of this subgroup [9,14]. However, the biological differences between HER2-low and HER2-zero (IHC 0) tumors, and how these differences affect responses to standard therapies—particularly CDK4/6 inhibitors—remain incompletely understood. Current evidence suggests that HER2-low disease may represent a biologically distinct subgroup within the broader heterogeneous HER2-negative population [15,16]. Therefore, determining the prognostic or predictive significance of HER2 expression levels in HR+/HER2− metastatic patients receiving CDK4/6 inhibitors holds important clinical value.

In our study, the prevalence of HER2-low tumors was 39.5%, which is consistent with the 31–64% range reported in the literature [17,18]. This wide range in previous studies may be attributed to technical variations in HER2 IHC scoring between laboratories and pathologists, temporal and spatial heterogeneity between primary and metastatic samples, as well as differences in patient selection criteria.

Regarding treatment response, no significant differences were observed between the HER2-low and HER2-zero groups in terms of objective response rate (ORR: 75.4% vs. 72.7%) or disease control rate (DCR: 91.0% vs. 88.8%). These findings are consistent with the reports by Shao et al. and Yildirim et al., supporting the notion that low-level HER2 expression does not meaningfully influence response to endocrine therapy combined with CDK4/6 inhibitors [19,20]. In contrast, Sharaf et al. reported a higher ORR in patients with HER2-zero disease (52.0% vs. 39.4; *p* = 0.005) [21]; however, this discrepancy is likely attributable to substantial differences in patient selection and treatment characteristics. In our study, patients who had received prior chemotherapy in the metastatic setting were excluded, CDK4/6 inhibitors were predominantly administered in earlier lines in combination with aromatase inhibitors, and the rate of visceral metastasis was lower (36.5%). Conversely, the cohort reported by Sharaf et al. included a higher proportion of patients treated in later lines, more frequent use of ribociclib in combination with fulvestrant, and a greater burden of visceral disease (52.5%). These factors may have disproportionately affected response outcomes, particularly in the HER2-low subgroup. Taken together, these data suggest that in relatively homogeneous, endocrine-sensitive populations, HER2-low expression does not represent a determinant of short-term response to CDK4/6 inhibitor–based therapy.

A similar pattern was observed in survival outcomes. Among patients receiving first-line endocrine therapy plus CDK4/6 inhibitors, median progression-free survival (PFS) was 23.91 months in the HER2-low group and 25.16 months in the HER2-zero group, with no statistically significant difference between the two (log-rank *p* = 0.785). Clinicopathological factors known to influence PFS—including the presence of visceral metastases (36.5%), de novo metastatic disease (60.8%), hormone receptor expression levels, Ki-67 index, and the specific CDK4/6 inhibitor used—were well balanced across groups, and HER2 status did not emerge as an independent predictor of disease progression in multivariable Cox regression analysis. These results align with prior studies by Tarantino et al. and Yildirim et al., which similarly found no significant association between HER2 expression and PFS in patients treated with CDK4/6 inhibitor–based regimens [20,22]. In contrast, shorter PFS in HER2-low disease has been reported by Zattarin et al. and Sharaf et al. [21,23]; however, those studies included cohorts with higher rates of visceral metastasis, greater baseline disease burden, more frequent use of CDK4/6 inhibitors beyond the first-line setting, and longer follow-up durations, all of which may have accentuated potential prognostic differences.

Overall survival (OS) analyses further supported these observations. Median OS did not differ significantly between the HER2-low and HER2-zero groups (49.54 vs. 53.12 months; log-rank *p* = 0.649). Key clinicopathological variables affecting OS were similarly balanced between groups, and HER2 status was not identified as an independent prognostic factor in multivariable analysis. These findings are concordant with previous reports by Carlino et al., Wu et al., and Sottotetti et al., which demonstrated no clinically meaningful impact of HER2-low status on OS in the context of CDK4/6 inhibitor–based therapy [24,25,26]. In contrast, they differ from the findings of Zattarin et al. [23], whose cohort was characterized by longer follow-up, a higher number of subsequent treatment lines, and heterogeneity in HER2 assessment and post-progression management, factors that may have contributed to the observed OS disadvantage in HER2-low disease. Collectively, these results indicate that under the current first-line treatment strategy combining endocrine therapy with CDK4/6 inhibitors, low HER2 expression does not demonstrate independent prognostic value in a relatively homogeneous, endocrine-sensitive population. However, its potential relevance in subsequent lines of therapy or in alternative treatment combinations cannot be excluded and warrants further investigation.

Beyond its current prognostic implications, HER2-low breast cancer has attracted growing interest as a potential therapeutic target. Recent advances in engineered anti-HER2 drug delivery nanosystems aim to enhance treatment efficacy even in tumors with low or heterogeneous HER2 expression, highlighting the evolving role of HER2 biology beyond conventional immunohistochemical classification [27]. Although our study did not demonstrate a prognostic impact of HER2-low status under current CDK4/6 inhibitor–based therapies, these emerging approaches suggest a potential predictive relevance in future personalized treatment strategies.

## 5. Strengths and Limitations

Key strengths of this study include the multicenter design, the relatively large and clinically homogeneous cohort treated uniformly with first-line endocrine therapy plus CDK4/6 inhibitors, and the balanced distribution of major clinicopathological variables between HER2-low and HER2-zero groups. However, several limitations should be acknowledged. The retrospective design introduces the potential for residual confounding, and the absence of centralized pathology review as well as the lack of HER2 reassessment in metastatic lesions may have led to misclassification and may not fully capture the spatiotemporal heterogeneity of HER2 expression. In addition, data on disease burden, performance status, comorbidities and prior adjuvant endocrine therapy were not consistently available. Finally, the relatively limited follow-up duration may have influenced survival estimates, highlighting the need for prospective studies with standardized biomarker assessment.

## 6. Conclusions

In this multicenter retrospective cohort of patients with HR-positive/HER2-negative metastatic breast cancer treated with first-line endocrine therapy plus CDK4/6 inhibitors, low-level HER2 expression did not significantly influence treatment response, progression-free survival, or overall survival. These findings suggest that HER2-low status does not represent a prognostic or predictive biomarker in this relatively homogeneous, endocrine-sensitive population receiving CDK4/6 inhibitor-based therapy. Further prospective studies with standardized HER2 assessment and longer follow-up are warranted to clarify the biological and clinical implications of HER2-low expression in this setting.

## Figures and Tables

**Table 1:** Comparison of baseline clinical and demographic between HER2-zero and HER2-low groups.

Characteristic	HER2-Zero (*n* = 187), *n* (%)	HER2-Low (*n* = 122), *n*(%)	*p* Value
Age, years, median (range)	53 (26–86)	53 (27–90)	0.918
Age group			0.458
<65	139 (74.3)	86 (70.5)	
≥65	48 (25.7)	36 (29.5)	
Menopausal status			0.670
Premenopausal	54 (28.9)	38 (31.1)	
Postmenopausal	133 (71.1)	84 (68.9)	
Histology			0.761
Ductal	135 (72.2)	90 (73.8)	
Others	52 (27.8)	32 (26.2)	
Estrogen receptor			0.405
High > 50	170 (91)	114 (93.5)	
Low ≤ 50	17 (9)	8 (6.5)	
Progesteron receptor			0.295
High ≥ 20	137 (73.3)	96 (78.7)	
Low < 20	50 (26.7)	26 (21.3)	
Ki67 index %			0.076
High ≥ 20	125 (66.8)	94 (77)	
Low < 20	62 (33.2)	28 (23)	
Disease status			0.436
De novo metastatic	115 (61.5)	73 (59.8)	
Recurrent metastatic	72 (38.5)	49 (40.2)	
Site of metastasis			0.413
Visceral	65 (34.8)	48 (39.4)	
Nonvisceral	122 (65.2)	74 (60.6)	

**Table 2:** Treatment Response Rates in HER2-low and HER2-0 Groups.

Parameter	HER2-Low (%)	HER2-0 (%)	Odds Ratio (95% CI)	*p*-Value
Objective Response Rate (ORR)	(75.4%)	(72.7%)	1.182 (0.790–1.769)	0.415
Disease Control Rate (DCR)	(91%)	(88.8%)	1.290 (0.580–2.871)	0.532

**Table 3:** Medyan PFS and OS in HER2-low and HER2-0 Groups.

Parameter	HER2-Low Months (95% CI)	HER2-0 Months (95% CI)	*p*-Value
Progression-Free Survival (PFS)	23.91 (18.71–29.12)	25.16 (17.64–32.68)	0.785
Overall Survival (OS)	49.54 (41.82–57.26)	53.12 (44.69–61.55)	0.649

**Table 4:** Prognostic variables for progression-free survival in univariate analysis and multivariate analysis.

	Univariate Analysis		Multivariate Analysis	
Variables	HR (95% CI for HR)	*p* Value	HR (95% CI for HR)	*p* Value
HER2-0 vs. HER2-low	1.04 (0.74–1.46)	0.785		
Age ≥ 65 years vs. <65 years	1.02 (0.71–1.48)	0.887		
De novo vs. recurrent metastatic	1.46 (1.05–2.02)	0.023	1.32 (0.94–1.87)	0.103
ER > 50% vs. ER ≤ 50%	1.59 (0.93–2.71)	0.09		
PR ≥ 20% vs. PR < 20%	1.69 (1.19–2.42)	0.003	1.58 (1.09–2.30)	0.016
Ki67 < 20% vs. Ki67 ≥ 20%	1.98 (1.29–3.04)	0.002	1.73 (1.11–2.68)	0.015
Non-visceral vs. Visceral disease	1.59 (1.14–2.22)	0.006	1.62 (1.15–2.28)	0.006

**Table 5:** Prognostic variables for overall survival in univariate analysis and multivariate analysis.

	Univariate Analysis		Multivariate Analysis	
Variables	HR (95% CI for HR)	*p* Value	HR (95% CI for HR)	*p* Value
HER2-0 vs. HER2-low	1.11 (0.70–1.76)	0.649		
Age ≥ 65 years vs. <65 years	1.56 (0.97–2.50)	0.064	1.57 (0.98–2.51)	0.06
De novo vs. recurrent metastatic	1.12 (0.71–1.77)	0.607		
ER > 50% vs. ER ≤ 50%	1.61 (0.79–3.29)	0.183		
PR ≥ 20% vs. PR < 20%	1.51 (0.93–2.46)	0.094		
Ki67 < 20% vs. Ki67 ≥ 20%	1.59 (0.91–2.77)	0.103		
Non-visceral vs. visceral disease	1.74 (1.10–2.74)	0.017	1.74 (1.10–2.75)	0.017

## Data Availability

The original contributions presented in this study are included in the article. Further inquiries can be directed to the corresponding author.
